# 氨基酸型亲水作用色谱固定相的制备及其色谱性能评价

**DOI:** 10.3724/SP.J.1123.2025.04015

**Published:** 2025-07-08

**Authors:** Gaigai XU, Yang YI, Pingping LIU, Wenfen ZHANG

**Affiliations:** 1.郑州轻工业大学食品与生物工程学院，河南 郑州 450002; 1. School of Food and Bioengineering，Zhengzhou University of Light Industry，Zhengzhou 450002，China; 2.中国烟草总公司郑州烟草研究院，河南 郑州 450001; 2. Zhengzhou Tobacco Research Institute of CNTC，Zhengzhou 450001，China; 3.郑州大学化学学院，河南 郑州 450001; 3. College of Chemistry，Zhengzhou University，Zhengzhou 450001，China

**Keywords:** L-羟基脯氨酸, L-脯氨酸, 氨基酸固定相, 亲水相互作用液相色谱, L-hydroxyproline, L-proline, amino acid-functionalized stationary phase, hydrophilic interaction liquid chromatography （HILIC）

## Abstract

以亲水性L-羟基脯氨酸和L-脯氨酸为改性剂，采用连续固液反应，成功制备了两种氨基酸型亲水相互作用液相色谱（HILIC）固定相，并通过红外光谱分析、热重分析、元素分析对其结构进行了系统表征。结果表明，两种功能化色谱固定相制备成功，键合量分别为0.193 mmol/g和0.178 mmol/g，均具有良好的热力学稳定性。在HILIC模式下，以磺胺、杂环胺、核苷、植物生长调节剂、黄酮、胺类物质为溶质，考察了两种固定相的色谱分离性能，结果表明，L-脯氨酸固定相和L-羟基脯氨酸固定相与分析物之间均存在亲水、*π-π*等相互作用，并且L-羟基脯氨酸固定相还存在氢键相互作用。L-羟基脯氨酸固定相对磺胺、植物生长素、黄酮等极性小分子具有良好的分离效果，证明该固定相可用于极性化合物分离。该研究拓展了氨基酸在色谱分离领域的应用，也为HILIC固定相的开发提供了新思路。

## Abstract (en)

To overcome current limitations in polar compound separation and better understand hydrophilic interaction liquid chromatography （HILIC） retention mechanisms， we designed and synthesized two novel amino acid-functionalized stationary phases using highly hydrophilic L-hydroxyproline and L-proline as modifiers through a continuous solid-liquid reaction method. The synthesized stationary phases were thoroughly characterized using Fourier-transform infrared spectroscopy （FT-IR）， thermogravimetric analysis （TGA）， and elemental analysis. Comparative elemental analysis revealed a substantial increase in carbon （C）， hydrogen （H）， and nitrogen （N） contents in both L-hydroxyproline-functionalized （L-OH-PSil） and L-proline-functionalized （L-PSil）stationary phases relative to the cyanuric chloride-bonded aminopropyl silica gel （TCT-Sil） intermediate， confirming successful functionalization. Quantitative analysis demonstrated distinct ligand densities for each phase， with L-OH-PSil exhibiting a higher loading （0.193 mmol/g） compared to L-PSil （0.178 mmol/g）. Thermal stability assessments indicated both materials maintained excellent structural integrity across a wide temperature range （20-600 ℃）， as evidenced by TGA results. To explore the chromatographic separation performance of the prepared L-OH-PSil and L-PSil stationary phases， sulfonamides were selected as solutes， and preliminary chromatographic separation investigations were conducted. The sulfonamide compounds exhibited excellent separation efficiency on both stationary phases， with retention behavior following consistent elution orders strongly correlated with analyte polarity. This observed retention pattern strongly suggested hydrophilic interactions constituted the predominant retention mechanism between the amino acid-functionalized stationary phases and sulfonamide analytes. Further supporting this conclusion， a systematic decrease in retention factors （lg *k* values） with increasing aqueous content in the mobile phase was observed as a characteristic feature of HILIC. Under optimized HILIC conditions， we further systematically evaluated the separation performance of both stationary phases using heterocyclic amines and nucleosides as model analytes， with direct comparison to a commercial Hypersil NH₂ column. Both custom phases exhibited exceptional column efficiency， with L-OH-PSil achieving 11 582.87 theoretical plates for 2-amino-3-methyl-9*H*-pyrido［2，3-b］indole （MeAαC） compared to 8 661.45 for L-PSil， while maintaining excellent performance across diverse analyte classes including plant growth hormones， flavonoids， and amines. The L-OH-PSil phase demonstrated superior chromatographic performance relative to both its L-PSil counterpart and the commercial NH₂ column. This superiority is attributable to its unique bifunctional design incorporating two hydroxyl groups， which combine the advantageous features of amino acid and diol-based stationary phases. This structural characteristic enables multiple synergistic interaction mechanisms， including *π-π* stacking， enhanced ion-exchange capacity， and additional hydrogen bonding sites， collectively yielding improved selectivity for polar small molecules. To further evaluate the chromatographic performance of the amino acid-based stationary phases， we investigated the effects of flow rate and column temperature using nucleosides and heterocyclic amines as model analytes. Remarkably， baseline separation was maintained even at elevated flow rates， demonstrating the robustness of both phases under high-throughput conditions. Temperature-dependent studies revealed that retention times exhibited only minor decreases or remained stable with increasing column temperature， suggesting minimal thermal effects on retention behavior. Van’t Hoff analysis yielded excellent linear correlations （*r*²=0.992 9-0.999 7） for all tested analytes， confirming that the retention mechanism remains unchanged across the studied temperature range while indicating an exothermic separation process. Method validation confirmed the reliability of the developed system， with chromatographic peaks maintaining excellent shape and retention time stability across varying analyte concentrations. The relative standard deviations （RSDs） of retention times for six nucleosides ranged from 0.29% to 0.59%， underscoring the outstanding operational stability and analytical reproducibility of the L-OH-PSil stationary phase. These results collectively demonstrate the robustness of the amino acid-functionalized stationary phases under varying chromatographic conditions， further supporting their potential for practical applications in polar compound analysis. These results indicate that the L-OH-PSil stationary phase has excellent potential for broad applications in pharmaceutical analysis， environmental monitoring， and bioanalytical separations.

高效液相色谱法（HPLC）作为现代分离分析技术的核心手段，在化学、医学、生命科学及环境科学等诸多领域发挥着不可替代的作用^［1-4］^。依据溶质-固定相相互作用机理的差异，HPLC主要分离模式包括：反相液相色谱（RPLC）^［5］^、亲水相互作用液相色谱（hydrophilic interaction liquid chromatography， HILIC）^［6，7］^、离子交换色谱^［8］^以及尺寸排阻色谱^［9］^。其中，RPLC^［5，10］^虽对非极性/弱极性化合物展现出卓越的分离选择性和重现性，但其对强极性及离子型化合物的保留能力与分离选择性存在明显局限。相较而言，HILIC^［11，12］^采用高有机相/低水相流动相体系，无需离子对试剂即可实现对极性化合物的有效保留，同时与质谱^［13-15］^和蒸发光散射检测器^［16］^具有良好的兼容性。作为HPLC系统的核心组件，色谱固定相通过其功能基团调控多元分子相互作用，不仅决定分离模式的选择，更直接影响分析物的保留行为^［17，18］^。因此，新型功能化固定相的设计合成^［1，19-21］^及对其分离机理的深入探究，始终是色谱学科发展的前沿研究方向之一。

极性官能团（如氨基、酰胺基和磺酸基）功能化的HILIC固定相^［22，23］^已被广泛应用于氨基酸^［24］^、多肽、核苷等生物分子^［25，26］^的分离分析。然而，现有固定相在亲水性、选择性和重现性等方面存在诸多局限，特别是分离效率不足，严重制约了其实际应用。氨基酸独特的结构特性可以与溶质分子产生多重分子相互作用（如氢键、离子交换和疏水作用等）^［27］^，在色谱固定相设计中展现出显著优势^［28，29］^。尽管如此，氨基酸功能化固定相仍存在合成路线复杂、化学稳定性不足、选择性与通量之间的平衡难以调控等问题。

基于此，本研究以水溶性L-羟基脯氨酸和L-脯氨酸为亲水功能基团，以三聚氯氰为连接手臂，采用连续固液反应制备了两种氨基酸型HILIC固定相，并采用红外光谱分析、热重分析和元素分析对固定相的结构和性质进行了表征。结果表明所制备固定相的合成步骤简单、具有较高的稳定性和良好的重复性。实验选取磺胺、杂环胺、核苷、植物生长素、黄酮、胺类等极性分子对所制备固定相的色谱分离性能和分离机理进行了系统研究。结果表明，所制备的两种色谱固定相基于其丰富的活性位点，可以与溶质分子间发生亲水作用、*π-π*作用、氢键作用等多重相互作用力，为氨基酸型HILIC固定相的商业开发和应用提供了一定的方法和实验基础。

## 1 实验部分

### 1.1 仪器与试剂

Agilent 1260高效液相色谱仪（美国Agilent公司）；Flash EA 1112元素分析仪（美国Thermo公司）；Bruker Vector 22红外光谱仪（德国Bruker公司）；STA 409 PC热重分析仪（德国Netzsch公司）；LX-2纯水机（美国Millipore公司）；Xk96-B涡旋仪（姜堰市新康医疗器械有限公司）；KQ-2200DA超声波清洗器（昆山市超声仪器有限公司）；150 mm×4.6 mm不锈钢柱管（郑州英诺生物科技有限公司）；CGY-100B色谱装柱机（深圳市正大流体机电设备有限公司）。

多孔硅胶（粒径5 μm，孔径10 nm，比表面积300 m^2^/g）购自中国科学院兰州化学物理研究所；L-羟基脯氨酸（99%）、L-脯氨酸（99%）和*N，N*-二异丙基乙胺（DIPEA，99%）均购自上海阿拉丁生化科技股份有限公司；三聚氯氰（99%）和硅烷化试剂3-氨丙基三乙氧基硅烷（KH-550，99%）均购自上海晶纯生化科技股份有限公司；磺胺类、杂环胺类、核苷类、植物生长素类、黄酮类、胺类化合物（分析纯）均购自上海麦克林生化科技有限公司；色谱纯甲醇、乙腈购自美国默克公司。其他药品无特殊说明均为分析纯。

### 1.2 中间体三聚氯氰键合氨丙基硅胶的制备

#### 1.2.1 硅胶的活化

称取6.0 g多孔硅胶浸入120 mL盐酸溶液（浓盐酸和水的体积比为1∶3）中，浸泡24 h，机械搅拌下回流12 h除去金属离子，用G5砂芯漏斗抽滤，高纯水洗涤至中性，100 ℃真空干燥，即得活化硅胶（SiO_2_）。

#### 1.2.2 3-氨丙基三乙氧基硅烷键合硅胶的制备

取10 mL KH-550于250 mL三口烧瓶中，并加入100 mL新蒸的无水甲苯混匀，机械搅拌下缓慢加入5 g活化硅胶，N_2_保护下升温至回流状态，反应24 h后，冷却，过滤，然后依次用甲苯、丙酮、高纯水、丙酮各洗涤3次，80 ℃真空干燥，即得3-氨丙基三乙氧基硅烷键合硅胶（3-aminopropyltriethoxy-silane-bonded silica gel， APS-Sil）。

#### 1.2.3 三聚氯氰键合氨丙基硅胶的合成

称取1.5 g三聚氯氰于250 mL三口烧瓶中，加入70 mL已除水甲醇；称取3 g APS-Sil于恒压滴液漏斗中，并加入100 mL已除水甲醇和5 mL DIPEA，在冰浴条件下，将其滴加到烧瓶中，机械搅拌反应9 h，停止反应，用G5砂芯漏斗过滤，依次用高纯水、甲醇和丙酮洗涤，70 ℃真空干燥12 h，即得三聚氯氰键合氨丙基硅胶（cyanuric chloride-bonded aminopropyl silica gel， TCT-Sil）。

#### 1.2.4 氨基酸钠的合成

称取1.5 g氢氧化钠、1.5 g氨基酸分别溶于甲醇中，搅拌下将氢氧化钠甲醇溶液缓慢滴加到氨基酸甲醇溶液中，滴加完后，再搅拌反应30 min，停止搅拌，旋蒸除去溶剂，80 ℃真空干燥9 h，即得氨基酸钠（L-羟基脯氨酸钠和L-脯氨酸钠）。

### 1.3 亲水型氨基酸固定相的制备

将合成的TCT-Sil和1 g氨基酸钠加入250 mL三口烧瓶中，并向其中加入150 mL甲醇和4 mL DIPEA。先在50 ℃反应2 h，再回流反应8 h。停止反应，用G5砂芯漏斗过滤，依次用高纯水、甲醇和丙酮洗涤，80 ℃真空干燥12 h。得到以三聚氯氰为连接手臂的氨基酸键合硅胶固定相，即L-羟基脯氨酸键合硅胶固定相（L-hydroxyproline-functionalized stationary phase， L-OH-PSil）和L-脯氨酸键合硅胶固定相（L-proline-functionalized stationary phase， L-PSil）。合成路线见图1。

**图1:**
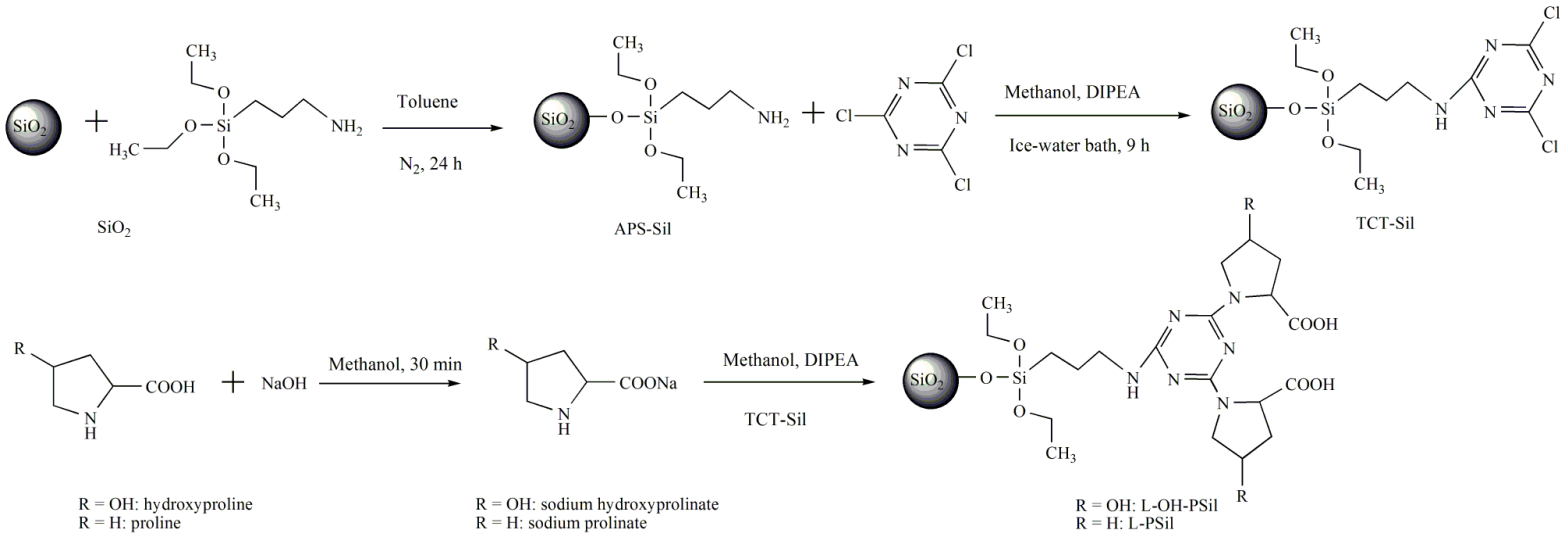
氨基酸型HILIC固定相的合成路线

### 1.4 色谱柱的填装

以四氯化碳作为匀浆液，甲醇作为顶替液，在装柱压力25 MPa下装填10 min，40 MPa下装填5 min，将所制备的固定相填料装入洗净的不锈钢柱管（150 mm×4.6 mm）中，30 min后，缓慢地降压，停泵。将装满填料的柱子柱头压平，然后装上滤片和柱接头，标上柱方向、填料、柱尺寸和装柱日期。在0.2 mL/min的流速下用甲醇冲洗24 h，使用前再用流动相平衡。

### 1.5 色谱条件

分离磺胺、杂环胺、核苷、植物生长调节剂、黄酮、胺类的流动相均采用乙腈-水。磺胺的波长为270 nm，其余均为254 nm，流速均为1.0 mL/min，采用等度洗脱；柱温均为30 ℃。

## 2 结果与讨论

### 2.1 L-OH-PSil和L-PSil**的表征**

采用红外光谱分析、元素分析、热重分析对所制备的固定相进行了表征。由表1可以看到，与中间体TCT-Sil相比，固定相L-OH-PSil和L-PSil中的C、H、N的含量有较为明显的增加，表明氨基酸已成功键合到APS-氨丙基硅胶上。以碳元素的含量（减去APS-三聚氯氰的碳含量）计算得到固定相L-OH-PSil和L-PSil的键合量分别为0.193 mmol/g和0.178 mmol/g。图2a为L-OH-PSil和L-PSil的红外光谱图，相对于SiO_2_，APS-Sil在2 933 cm^-1^和2 884 cm^-1^附近出现了甲基与亚甲基的C-H伸缩振动吸收峰，表明3-氨丙基三乙氧基硅烷成功键合到活化的硅胶上。与APS-Sil相比，TCT-Sil在1 595 cm^-1^处有C=N双键吸收峰，表明三聚氯氰成功键合到APS上。在纯硅胶、氨基酸型固定相中含有最多的键是Si-O键（1 110 cm^-1^），含量最多的基团是Si-OH（3 500~3 200 cm^-1^），805~465 cm^-1^处是硅胶介孔结构中Si-O键的特征吸收峰。然而硅胶的特征红外光谱吸收峰掩盖了固定相上C-C键的伸缩振动峰（1 250~1 140 cm^-1^）和醇羟基及N-H键的伸缩振动峰（3 500~3 300 cm^-1^），只能从氨基酸固定相红外光谱图中看到C-H键的伸缩振动峰2 933 cm^-1^和C=N键的吸收峰1 595 cm^-1^。由红外光谱表征图和元素分析数据可知，两种氨基酸均成功键合至硅胶上。由图2b可知，L-OH-PSil和L-PSil的失重温度均在180 ℃以上，且在20~600 ℃范围内，两种固定相具有较好的热稳定性。

**表1:** 元素分析结果

Analyte	Elemental contents/%			Surface coverage/（mmol/g）
	N	C	H	
APS-Sil	1.558	5.570	1.282	0.928
TCT-Sil	2.831	6.482	1.227	0.253
L-OH-PSil	2.885	8.801	1.520	0.193
L-PSil	2.888	8.614	1.320	0.178

APS-Sil： 3-aminopropyltriethoxysilane-bonded silica gel； TCT-Sil： cyanuric chloride-bonded aminopropyl silica gel； L-OH-PSil： L-hydroxyproline-functionalized stationary phase； L-PSil： L-proline-functionalized stationary phase.

**图2:**
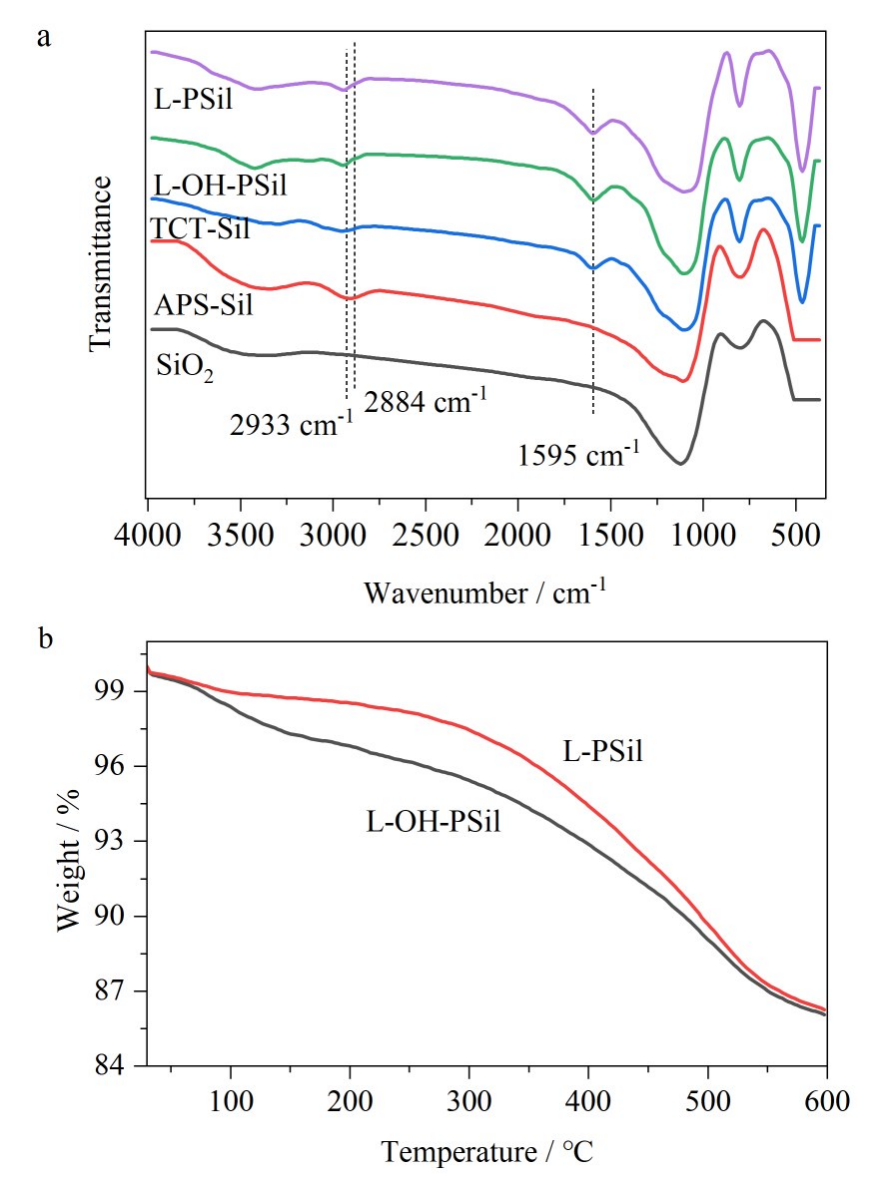
L-OH-PSil和L-PSil的表征

### 2.2 L-OH-PSil和L-PSil的色谱分离性能考察

为探究所制备L-OH-PSil和L-PSil的色谱分离性能，实验选取多种极性靶标为溶质，在HILIC模式下考察了其色谱分离行为，初步探究L-OH-PSil和L-PSil的色谱分离性能以及可能存在的分离机理，见图3。

**图3:**
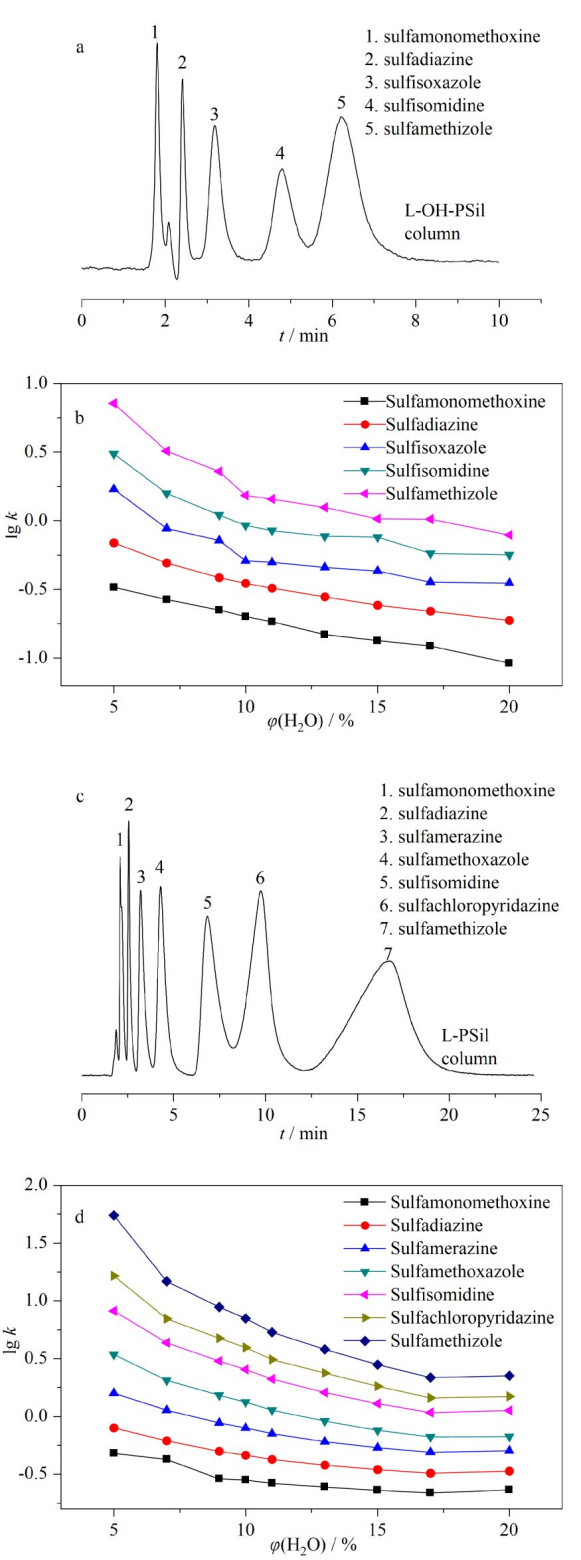
磺胺在L-OH-PSil和L-PSil上的分离性能和保留行为

首先，如图3a和3c所示，8 min内5种磺胺类物质在L-OH-PSil上实现了分离，20 min内实现了7种磺胺类物质在L-PSil上的分离。且同种磺胺类物质在两种氨基酸型固定相上的洗脱顺序一致且与化合物的极性成正比，由此说明两种氨基酸固定相与磺胺类物质间的亲水作用起主导作用。且随着流动相中水含量的增加（图3b和3d），磺胺类物质在两固定相上的lg *k*均逐渐减小，保留减弱，这与HILIC的分离机理一致。由此说明L-OH-PSil和L-PSil具有亲水色谱分离性能。

为进一步比较L-OH-PSil和L-PSil色谱柱的分离选择性差异，实验选取结构较为相似的8种杂环胺（图4a）和7种核苷（图4b）进行了色谱分离行为考察，并以Hypersil NH_2_色谱柱（150 mm×4.6 mm， 5 μm，美国Thermo公司）作为对比，结果见图5。可以看出，本研究制备的两种氨基酸功能化固定相对杂环胺和核苷类化合物的分离性能显著优于商品化氨基色谱柱；此外，在相同色谱条件下，L-OH-PSil可实现8种杂环胺的基线分离（图5a），而L-PSil对2-氨基二吡啶并［1，2-a：3′，2′-d］并咪唑（Glu-P-2）、2-氨基-1-甲基-6-苯基咪唑并［4，5-b］吡啶（PhIP）、9*H*-吡啶-［3，4-b］吲哚（Norharman）、2-氨基-1，6-二甲基咪唑并［4，5-b］吡啶（DMIP）和1-甲基-9*H*-吡啶-［3，4-b］吲哚（Harman）等化合物表现出共洗脱现象；类似地，L-OH-PSil可完全分离7种核苷（图5b），而L-PSil未能实现尿嘧啶与腺苷的基线分离。从分子结构角度分析，L-羟基脯氨酸固定相相较于L-脯氨酸固定相额外具有两个羟基官能团，这一结构特征使其兼具L-PSil和二醇基固定相双重色谱特性。除溶质在固定相-流动相界面“富水层”中的分配作用、*π-π*相互作用、离子交换作用等分子相互作用分离机制外，L-OH-PSil中羟基提供的氢键作用位点显著增强了其对极性小分子的分离选择性，从而实现了对8种杂环胺和7种核苷的高效分离。根据表2的色谱参数分析，两种氨基酸固定相在杂环胺分离中均表现出优异的柱效，其中MeAαC在L-OH-PSil和L-PSil上的理论塔板数分别达到11 582.87和8 661.45，证实了所开发固定相的高效分离性能。

**图4:**
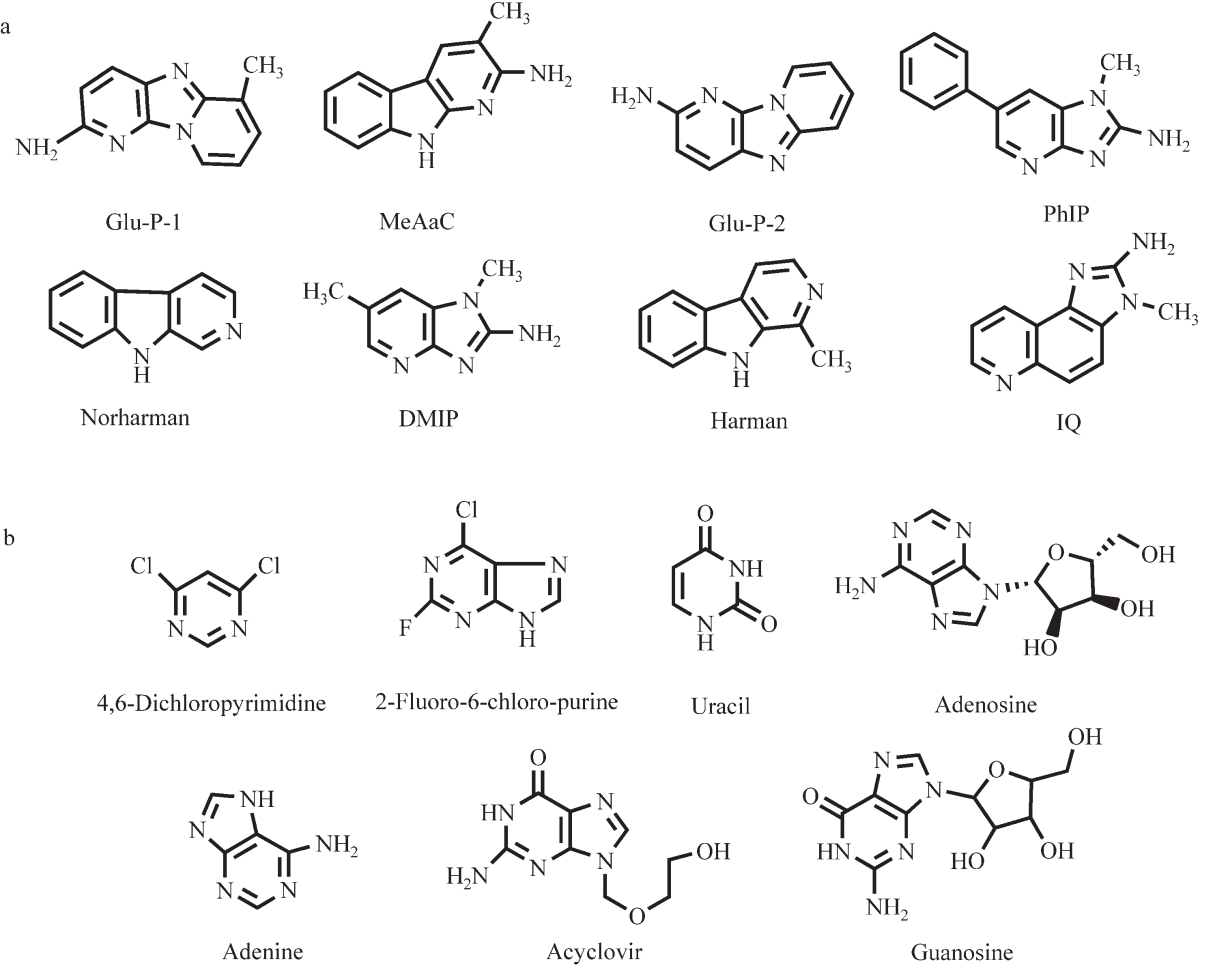
（a）8种杂环胺和（b）7种核苷的结构式

**图5:**
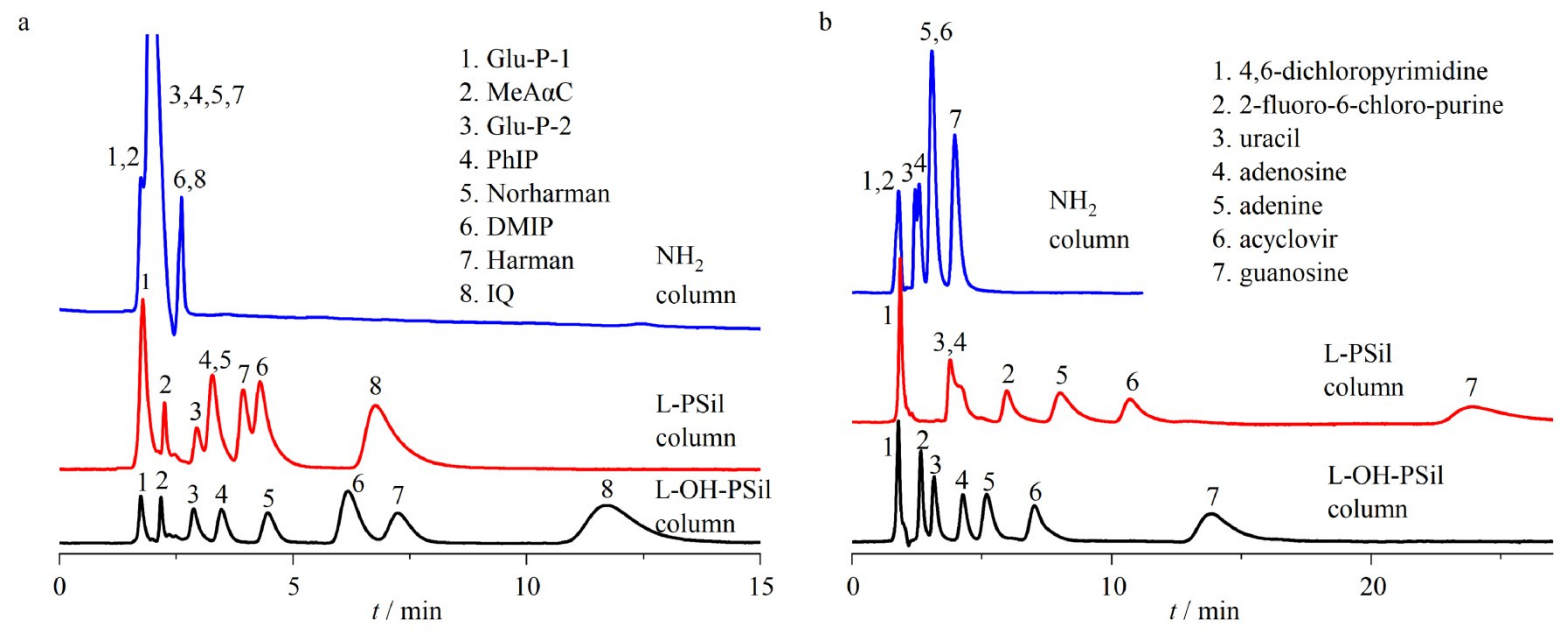
（a）8种杂环胺和（b）7种核苷在L-OH-PSil、L-PSil与Hypersil NH_2_柱的色谱图

**图6:**
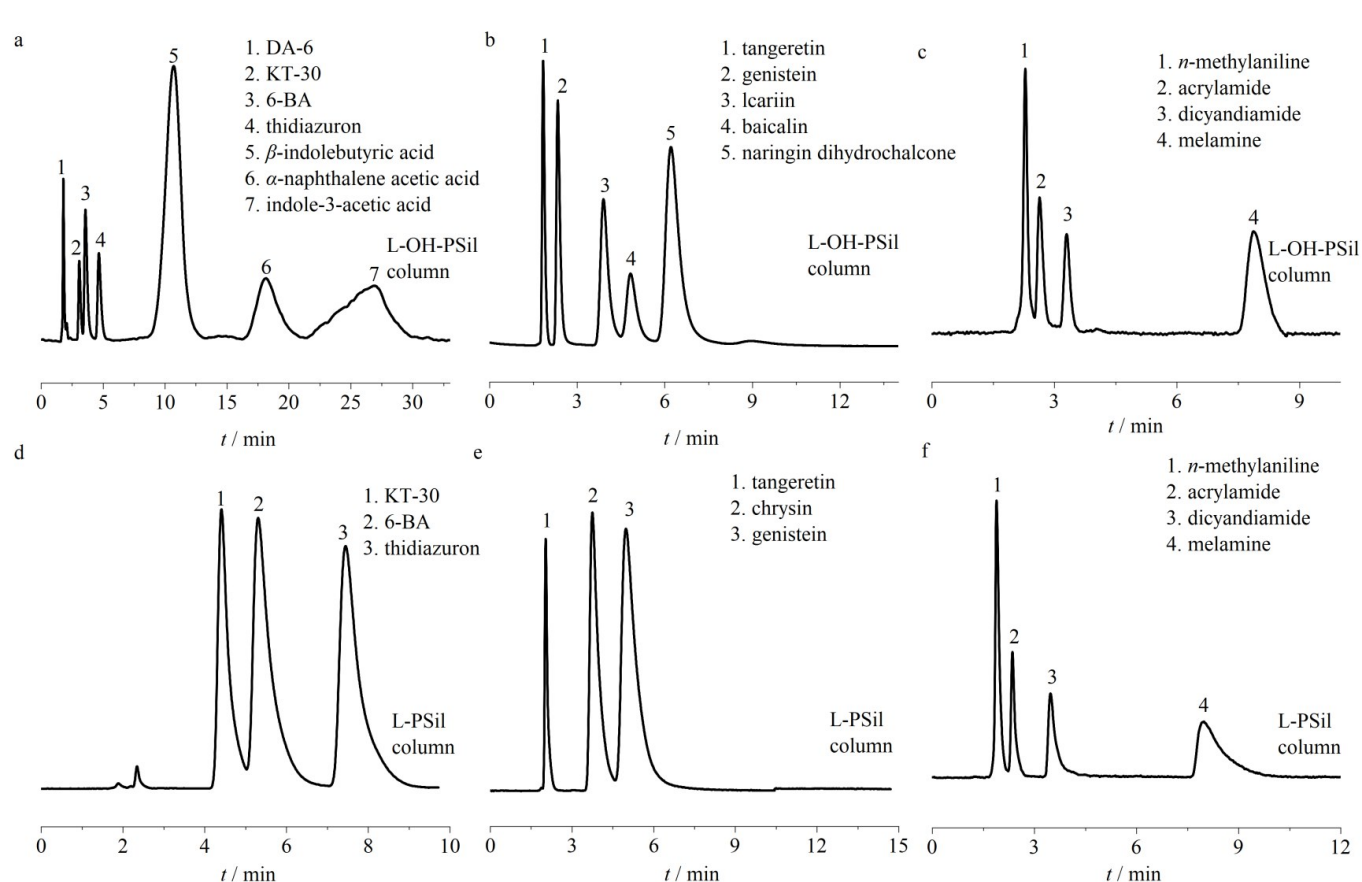
L-OH-PSil和L-PSil柱对亲水性物质的分离

**图7:**
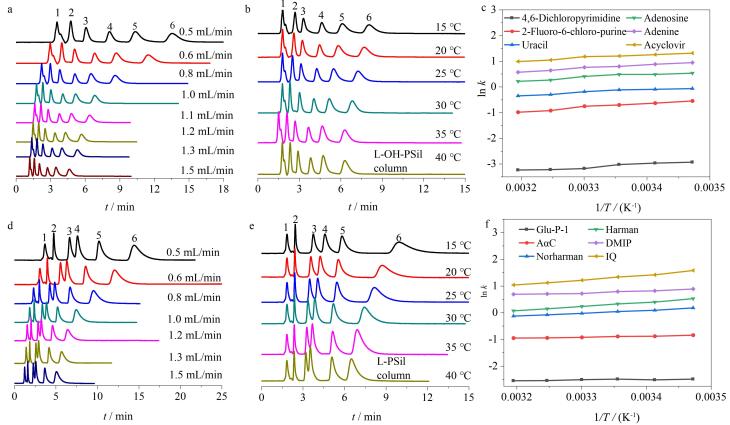
核苷在L-OH-PSil以及杂环胺在L-PSil柱上的分离性能

**表2:** 8种杂环胺在两种氨基酸型固定相上的理论塔板数

Column	Glu-P-1	MeAαC	Glu-P-2	PhIP	Norharman	DMIP	Harman	IQ
L-OH-PSil	3696.98	11582.87	3414.64	3889.27	4174.57	3701.19	3666.03	3299.56
L-PSil	2292.08	8661.45	3628.90	4376.20	4696.48	3377.73	3821.84	2950.15

为进一步探究两根色谱柱在亲水化合物分离中的通用性，选取植物生长素、黄酮类物质、胺类物质进行分离，结果见图6。

可以看出，7种植物生长调节剂、5种黄酮类物质、4种胺类物质在L-OH-PSil上均得到了较好的分离（图6a~c）；而对于L-PSil，只有3种极性较弱的植物生长素、3种黄酮类物质在L-PSil上实现了基线分离（图6d~f）。由此说明羟基等基团能够调控色谱固定相的保留行为，在实现化合物的高效分离中扮演着重要角色。

### 2.3 不同流速、柱温对色谱固定相分离性能的影响

色谱分离过程中，流速往往会影响各个溶质组分的保留时间及峰宽，从而对混合物的分离造成影响。实验分别考察了不同流速、柱温条件下核苷在L-OH-PSil及杂环胺在L-PSil上的分离效果，由图7a和图7d可知，随着流速的增加，核苷和杂环胺在两种固定相上的保留时间和分离度均逐渐降低。但即使在较高的流速条件下，核苷和杂环胺也能在两种氨基酸型固定相上进行较好的基线分离，这表明L-OH-PSil柱对核苷、L-PSil柱对杂环胺具有较好的分离选择性，可在较高的流速下分离极性物质。

实验考察了不同柱温（15~40 ℃）对两种色谱柱色谱分离的影响。由图7b和图7e可以看出，随着柱温的升高，6种核苷在L-OH-PSil柱的保留时间以及6种杂环胺在L-PSil柱的保留时间略微减少或基本不变，这说明核苷以及杂环胺在色谱柱上的分离并不是简单的放热过程，这说明核苷在L-OH-PSil色谱柱上的分离以及杂环胺在L-PSil色谱柱上的分离并不是简单的放热过程，可能由溶质分子与HILIC固定相间存在的分配作用、氢键作用、*π-π*作用等多重作用力引起；此外，由表3和表4可知，大部分核苷及杂环胺的分离选择性并不是单纯呈现上升或下降趋势，不同拐点可能是因为除亲水、疏水、分配作用外，固定相和靶标间还存在氢键作用等其他相互作用，导致趋势呈现复杂性。

**表3:** 不同温度下L-OH-PSil对6种核苷的选择因子

*T*/℃	4，6-Dichloropyrimidine	2-Fluoro-6-chloro-purine	Uracil	Adenosine	Adenine	Acyclovir
15	-	10.88	1.61	1.84	1.51	1.44
20	-	10.28	1.72	1.78	1.49	1.46
25	-	10.20	1.79	1.82	1.37	1.50
30	-	11.35	1.76	1.80	1.43	1.52
35	-	9.96	1.87	1.77	1.45	1.50
40	-	9.48	1.89	1.76	1.43	1.52

**表4:** 不同温度下L-PSil对6种杂环胺的选择因子

*T*/℃	Glu-P-1	AαC	Norharman	Harman	DMIP	IQ
15	-	5.19	2.77	1.41	1.43	2.01
20	-	5.12	2.64	1.36	1.51	1.83
25	-	4.94	2.54	1.33	1.59	1.73
30	-	4.86	2.46	1.30	1.62	1.64
35	-	4.96	2.37	1.26	1.74	1.51
40	-	4.94	2.29	1.22	1.86	1.41

由范特霍夫曲线（图7c，f）可以看出，6种核苷在L-OH-PSil柱和6种杂环胺在L-PSil柱上均呈现良好的线性关系，相关系数（*r* ^2^）分别为0.994 2~0.996 8和0.992 9~0.999 7（表5），表明温度变化并没有改变溶质在固定相上的保留机制。根据溶质保留因子（*k*）和柱温（*T，* K）符合的范特霍夫方程：ln *k*=-*ΔH*/*RT*+Δ*S*/*R*，研究核苷和杂环胺分别在两种氨基酸固定相分离过程中的热力学过程。由表5可知，6种核苷在L-OH-PSil上和6种杂环胺在L-PSil上的焓变均为负值，这表明核苷和杂环胺在两种固定相上的分离过程为放热过程。

**表5:** 核苷在L-OH-PSil、杂环胺在L-PSil柱上的热力学参数Δ*H*、Δ*S*和拟合曲线的相关系数

Column	Analyte	Δ*H*/（kJ/mol）	Δ*S*/（J/（mol·K））	*r* ^2^
L-OH-PSil	4，6-dichloropyrimidine	-10.54	-60.81	0.9959
	2-fluoro-6-chloro-purine	-13.31	-50.95	0.9961
	uracil	-8.91	-31.21	0.9953
	adenosine	-9.94	-29.76	0.9968
	adenine	-11.29	-31.22	0.9942
	acyclovir	-9.71	-22.63	0.9949
L-PSil	Glu-P-1	-1.85	-27.06	0.9961
	AαC	-3.33	-18.93	0.9978
	Norharman	-9.07	-30.08	0.9931
	Harman	-13.31	-42.29	0.9962
	DMIP	-5.93	-13.38	0.9997
	IQ	-16.08	-42.87	0.9928

为系统评价L-OH-PSil的稳定性，以6种核苷分子为模型分析物进行分析研究。改变5 μg/mL核苷混合标准溶液的进样体积分别为1、3、5、7、9、10 μL，考察不同进样量对色谱分离的影响，实验叠加色谱图（图8）显示，各分析物色谱峰形保持良好，保留时间稳定，相对标准偏差（RSD）为0.29%~0.59%，充分证实L-OH-PSil色谱柱具有优异的操作稳定性和分析重复性，完全满足定量分析要求。特别值得注意的是，保留时间RSD全部低于0.59%，表明该材料在色谱分离应用中具有显著优势。

**图8:**
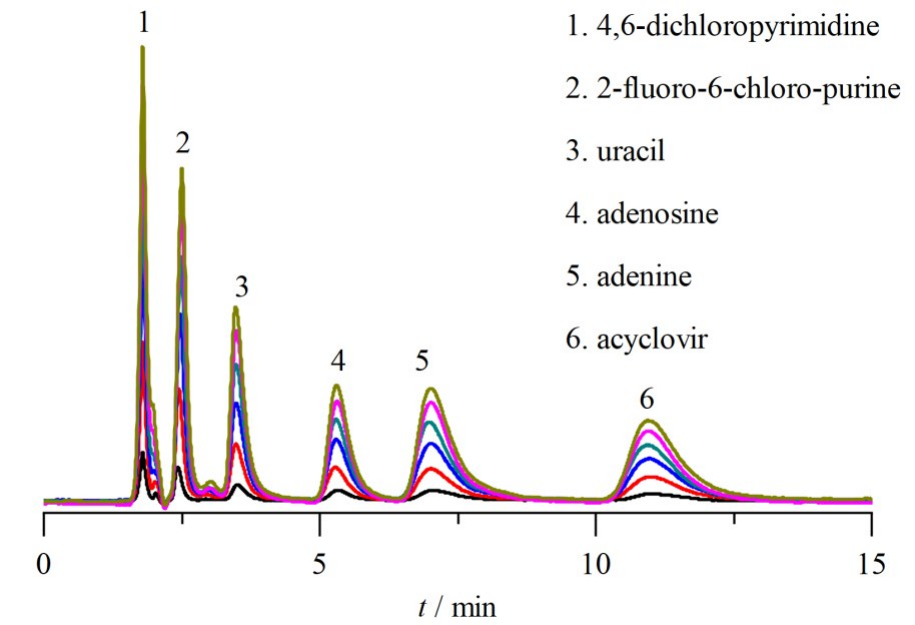
不同进样体积下L-OH-PSil分离6种核苷的稳定性

## 3 结论

本文以亲水性L-羟基脯氨酸和L-脯氨酸为改性剂，采用连续固液反应，成功制备了两种氨基酸型HILIC固定相（L-OH-PSil和L-PSil）。选取磺胺、杂环胺、核苷、植物生长调节剂、黄酮、胺类物质等极性小分子为溶质，对L-OH-PSil和L-PSil柱的分离性能和保留机制进行了考察，结果表明L-OH-PSil和L-PSil具有良好的亲水色谱分离性能，并且L-OH-PSil柱对极性化合物展现出卓越的分离选择性。L-OH-PSil柱表面丰富的活性位点在分离中具有调控亲水分配作用、氢键作用、*π-π*堆积、离子交换作用等多重相互作用的协同效应，显著提高了固定相的分离选择性。此外，L-OH-PSil柱在不同进样量下均表现出良好的重复性和稳定性。基于所制备固定相的活性位点以及良好的色谱性能，有望在药物分析、植物生理研究、代谢组学等领域展现出广阔的应用前景。

## References

[1] Zheng Q， Huang J， He Y， et al . ACS Appl Mater Interfaces， 2022， 14（7）： 9754 34990552 10.1021/acsami.1c20989

[2] Kobidze G， Sprega G， Daziani G， et al . J Chromatogr A， 2024， 1718： 464709 38350352 10.1016/j.chroma.2024.464709

[3] Zhang Q， Wu F， Liu J， et al . Chin Chem Lett， 2024： 110649

[4] Wang Q， Sun L， Wu H， et al . J Pharm Anal， 2022， 12（5）： 783 36320596 10.1016/j.jpha.2022.05.008 PMC9615578

[5] Kadlecová Z， Kalíková K， Tesařová E， et al . J Chromatogr A， 2022， 1681： 463473 36113338 10.1016/j.chroma.2022.463473

[6] Li Z， Li S， Zhang F， et al . Talanta， 2021， 231： 122340 33965018 10.1016/j.talanta.2021.122340

[7] Yu Z， Li Z， Zhang F， et al . J Chromatogr A， 2023， 1708： 464328 37666063 10.1016/j.chroma.2023.464328

[8] Yang Y， He H， Chen Y， et al . Chemosphere， 2024， 358： 142227 38704046 10.1016/j.chemosphere.2024.142227

[9] Panda S K， Al-Qunaysi T A， Al Taha M， et al . Fuel， 2021， 302： 120914

[10] Zhang Q-F， Xiao H-M， Zhan J-T， et al . Chin Chem Lett， 2022， 33（11）： 4746

[11] Fekete S， DeLano M， Harrison A B， et al . Anal Chem， 2022， 94（7）： 3360 35143179 10.1021/acs.analchem.1c05466

[12] Tang Z K， Wan H H， Li H， et al . Chinese Journal of Chromatography， 2023， 41（9）： 799 37712544 10.3724/SP.J.1123.2022.12014 PMC10507531

[13] Wang Y Q， Wang K， Ke Q Q， et al . Chinese Journal of Chromatography， 2024， 42（12）： 1164

[14] Gao W， Zhang F， Zhang S， et al . Sep Purif Technol， 2023， 305： 122426

[15] Feng J， Zhong Q， Kuang J， et al . Anal Chem， 2021， 93（45）： 15192 34739231 10.1021/acs.analchem.1c03905

[16] Zhao H， Lai C-J-S， Yu Y， et al . Int J Biol Macromol， 2020， 163： 476 32593759 10.1016/j.ijbiomac.2020.06.206

[17] Guo D M， Xia Y R， Rahman M U， et al . Chinese Journal of Chromatography， 2023， 41（12）： 1045 38093534 10.3724/SP.J.1123.2023.09028 PMC10719812

[18] Liu J， Wu F， Gan L， et al . Chinese Journal of Chromatography， 2023， 41（10）： 843 37875407 10.3724/SP.J.1123.2023.04021 PMC10598563

[19] Zhang T， Liang X， Wang L， et al . Chin Chem Lett， 2025， 36（1）： 109889

[20] Jiang Q， Xin X， Zhang S， et al . TrAC-Trends Anal Chem， 2024， 174： 117680

[21] Liu Q， Zhou K， Liu Y， et al . Anal Chim Acta， 2024， 1299： 342445 38499423 10.1016/j.aca.2024.342445

[22] Ma L， Liu S-M， Yao L， et al . J Chromatogr A， 2015， 1376： 64 25528071 10.1016/j.chroma.2014.12.002

[23] Shuang Y， Liao Y， Zhang T， et al . J Chromatogr A， 2020， 1619： 460937 32063276 10.1016/j.chroma.2020.460937

[24] Zhang S， Ee K H， Goh R M V， et al . Food Chem， 2025， 472： 142846 39827564 10.1016/j.foodchem.2025.142846

[25] Wu S， Li X， Zhang F， et al . Analyst， 2015， 140（12）： 3921 25946074 10.1039/c5an00570a

[26] Tsutsumiuchi K， Toyoshima T， Hasegawa F， et al . J Agric Food Chem， 2021， 69（13）： 3904 33761247 10.1021/acs.jafc.0c07948

[27] Liang M， Liu D， Nie Y， et al . Chin Chem Lett， 2022， 33（6）： 3123

[28] Nam D， Ji M， Kang C， et al . Anal Chem， 2024， 96（25）： 10219 38864836 10.1021/acs.analchem.4c00518

[29] Coppens S， Drinkwater G， Mussell C， et al . Alzheimer’s & Dementia， 2022， 18（S5）： e063409

